# Functional and Organic Foods in Enhancing the Oral and Dental Health and Hygiene—A Comprehensive Review

**DOI:** 10.1002/fsn3.71433

**Published:** 2026-02-05

**Authors:** Elahe Aleebrahim‐Dehkordi, Ahmad Sheibaninia, Abdolah Ghasemi Pirbalouti

**Affiliations:** ^1^ Systematic Review and Meta‐Analysis Expert Group (SRMEG) Universal Scientific Education and Research Network (USERN) Tehran Iran; ^2^ Medical Plants Research Center, Basic Health Sciences Institute Shahrekord University of Medical Sciences Shahrekord Iran; ^3^ Department of Orthodontics TeMS.C., Islamic Azad University Tehran Iran; ^4^ Department of Food Science and Technology, Institute of Food Science and Functional Foods TeMS.C., Islamic Azad University Tehran Iran

**Keywords:** dietary, functional foods, nutraceutical agents, oral and dental health, organic foods, oxidative stress

## Abstract

Maintaining oral and dental hygiene, it is significantly related to individuals' quality of life as well as systemic health. Despite its importance, oral health is often overlooked, especially among underserved populations who face barriers to accessing dental care. This neglect exacerbates health inequalities and leads to a cycle of poor health outcomes. Understanding these factors can lead to targeted interventions as well as appropriate public health strategies to improve access to dental care and promote healthy practices. Nutrition is recognized as an essential component in the prevention of oral and dental diseases. Based on these considerations, a better understanding of how diet, and in particular nutrients intake, influences the potential relationship between nutrition and oral and dental diseases including periodontal disease, oral mucositis, root canal infection, halitosis, etc. is needed. Among the innovations developed in the food market are products called functional foods, and recent trends in food demand indicate that consumers are increasingly aware of the link between diet and health. Functional and organic foods contain effective and non‐toxic bioactive compounds, which have potential benefits effect on health beyond their basic nutritional value. The relationship between food products with the approach of functional and organic foods has been investigated in various studies and it seems that the bioactive substances in herbs, fruits, vegetables, probiotics, etc. can control or treat the risks associated with oral diseases. A notable trend in the food industry is the technological advancement and development of an industrial chain that has shifted from functional agriculture to functional food, involving the selective use of plants, microorganisms, etc. as biological platforms for enriching nutrients in products with health‐promoting properties. This review aims to examine and introduce functional and organic foods and their application in dentistry for the prevention and treatment of oral and dental illnesses.

## Graphical abstract

Functional and organic foods contain effective and bioactive compositions. Organic foods and foods enriched with phytochemicals provide promising oral health. Functional foods and beverages in the diet can control and treat oral illnesses

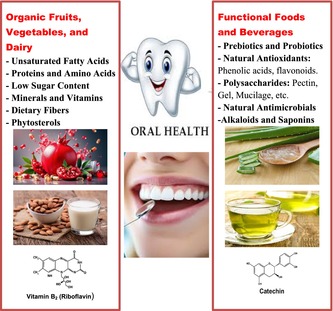

## 1 Introduction

Oral health is a very important indicator of overall health and quality of life. Based on the World Health Organization (WHO) report, oral health refers to a state in which a person is free from any chronic oral and facial pain, oral and throat cancer, oral infections and ulcers, periodontal (gum) disease, as well as tooth decay and tooth loss, etc. that affect a person's ability to bite, chew, smile, speak, and psychosocial well‐being. Oral and dental epidemiology has been changing globally in recent years. Among the important and major oral and dental diseases that affect the quality of life of people are tooth decay, periodontal disease, oral cancer, oral and dental trauma, etc., which can lead to severe pain, restlessness, infection, disruption of daily performance, and reduced the life (Huang et al. 2021).

Many developing and low‐income countries are unable to provide preventive and treatment services for oral diseases. Additionally, inadequate hygiene practices, unhealthy and high‐sugar diets, as well as tobacco and alcohol consumption are among the factors that contribute to the onset or progression of oral disorders. Most oral diseases are largely preventable if identified and controlled in their early stages; therefore, they can be treated, and their potential side effects avoided (Peres et al. 2020; Sridharan et al. 2020).

**FIGURE 1:**
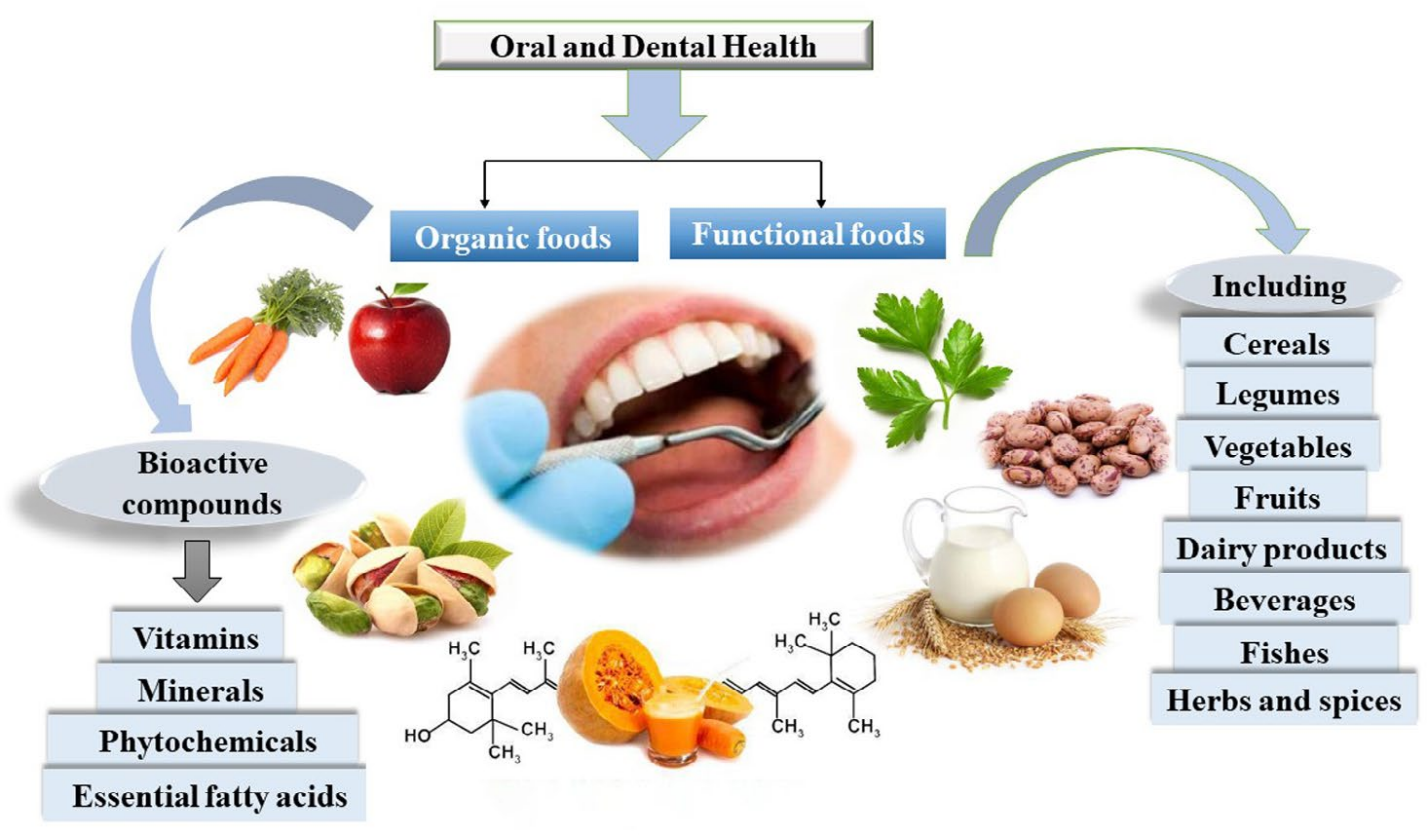
Infographic of promoting oral and dental health using functional and organic foods.

Today, functional and organic foods have been proposed as a solution to maintain nutritional health, and their production is increasing day by day. In the functional food industry, beneficial and active ingredients for health are added to foods, or harmful ingredients for health are removed or reduced. Currently, in intensive agricultural and husbandry systems, increasing productivity is highly dependent on the application of synthetic inputs such as pesticides, fungicides, antibiotics, fertilizers, hormones etc., The massive utilization of these synthesis inputs, in addition to human health hazards, causes negative impacts on the environmental ecosystems, the quantity and quality ingredients of agro‐foods (Kaviani Darani et al. 2025). As a result, a product is obtained that is available to the consumer as a health food. A diet rich in organic foods as well as containing vegetable and fish oils, food products enriched with probiotics and vitamins, and medicinal herbs, colorful vegetables and fruits of optimal quality in active ingredients like phenols, flavonoids, anthocyanins, fiber, etc. are among the super beneficial foods (Essa et al. 2023; Ghasemi Pirbalouti et al. 2013, 2014; Rahman et al. 2024; Rezaei and Ghasemi Pirbalouti 2019). Current trends in food demand indicate that consumer preferences are shifting towards foods that carry health‐related messages. Consumers believe that food affects their health status and want to use it as a tool to achieve or maintain health (Migliore et al. 2022). With the popularity of functional and organic foods, people are increasingly aware of the food quality as well as the health benefits associated with different foods. As a result, people's interest in consuming healthier foods and the demand for food products of natural origin, as well as organic foods is enhancing significantly (Kaviani Darani et al. 2025). Therefore, it is very necessary to understand and recognize functional foods, their actions on the body, and develop an innovative technological solution to meet these needs (Essa et al. 2023). This review aims to examine and introduce functional and organic foods, the position of these fruitful ingredients in the food industry, and their utilization in dentistry for the oral and dental diseases prevention and treatment (Figure 1).

## 2 Functional and Organic Foods

### 2.1 Functional Foods

Functional foods have a potentially beneficial impact on health beyond their basic nutritional value. These foods can promote health and reduce the risk of illnesses. The foods are gaining widespread popularity worldwide and are commonly referred to as “nutraceuticals” and “designer foods” (Essa et al. 2023). The functional foods have attracted significant research attention among scientists, investigators, and experts in this field over the decades, in terms of health areas of improvement and food technology (Sharma et al. 2024). Although the concept of functional foods has been defined many times, there is still no universally accepted definition of the term (Baker et al. 2022). More than 100 nutrition and related science experts (1995 to 1998), as part of the European Commission's Coordinated Action on Functional Food Sciences in Europe (FUFOSE) to develop a science‐based approach to concepts in applied food sciences [coordinated by the International Life Sciences Institute (ILSI)], reached a consensus on the definition of functional foods. The European consensus document defined functional foods as “foods that, beyond their nutritional effects, have a positive and satisfactory effect on one or more target functions in the body, in a way that can be associated with improved stage of health, and well‐being and/or reduced risk of disease” (Baker et al. 2022; Nazzaro et al. 2025), which has in fact been the most widely used definition of functional foods according to previous investigations. There are also alternative definitions among exist across food and nutrition institutions like the definition of functional foods by the “Institute of Medicine of the National Academy of Sciences (U.S.). Food and Nutrition Board” (in 1994) is defined as “any food or food ingredient that may provide health benefits beyond the traditional nutrients contained in it.” Similarly, the IFT (Institute of Food Technologists) (in 2005) defined functional foods as “foods that have health benefits beyond their basic nutritional.” The Functional Food Center (in 2014) defined functional foods as “natural or processed foods that contain biologically active compounds that in defined, effective, and non‐toxic amounts, have been shown to provide clinically, and documented health benefits, using specific biomarkers, for the prevention, management, or treatment of chronic diseases or their symptoms.” Despite all the different definitions offered, generally experts agree that functional foods actually have substances that provide health benefits beyond the primary nutritional components of the food's basic. Additionally, there have been many different attempts to provide a commonly agreed definition (Baker et al. 2022; Gur et al. 2018; Martirosyan et al. 2021). However, such a definition is not yet fully accepted, as other authors have excluded functional foods from products that naturally contain biologically active substances. In this regard, they claimed that foods can be defined as “functional foods” if they fall into the following categories: (1) products enriched with compounds that have a positive effect on health; (2) products that have been purified of anti‐nutritional compounds; (3) raw materials that have been improved or fortified/purified by changes in agricultural practices such as animal feeding, crop breeding, post‐harvesting technologies for improving the quality of herbs, vegetables, and fruits; (4) innovated foods with improved benefits for human health (Alongi and Anese 2021; Vlaicu et al. 2023).

### 2.2 Organic Foods

In recent years, the demand, consumption, and global marketplace for organic products have increased significantly, which could lead to meaningful and sustainable growth on the supply side of these products (Kaviani Darani et al. 2025). The term “organic” actually serves as a heuristic sign of superiority over conventional food. Naturally, these food products are not produced in harmony with all the factors considered in terms of pesticides and chemical‐free pesticides, and fertilizers, genetically modified organisms (GMOs), as well as irradiation (Gumber and Rana 2021; Nunes et al. 2021). The International Federation of Organic Agriculture Movements (IFOAM) even defined organic agriculture as “a production system that sustains the health of soils, ecosystems, and people”. According to definition of organic food by the United States Department of Agriculture, organic food is “a labeling term that indicates food or other agricultural products have been produced generally through approved methods”. In this regard, the ecological processes, biodiversity, and cycles adapted to local conditions are the main approaches that enhance resources, promote ecological balance, and conserve biodiversity (Chowdhury et al. 2021). Organically labeled food products derived from livestock, such as meat, chicken, eggs, dairy, and other products in organic agriculture and livestock systems are produced under defined animal health and welfare standards, restriction of certain antibiotics, growth hormones, pesticides, and fungicides, and the use of organic feed. From an environmental perspective, organic foods are considered inherently safe, in that the synthesis of chemical pesticides and fertilizers is not applied in the production of organic food products. Moreover, organic food products are not obtained from genetically modified organisms, nor as well as irradiation, industrial solvents, nor synthetic food additives are not used in these products (Gundala and Singh 2021; Schleiffer and Speiser 2022). Additionally, organic foods are healthier and more environmentally friendly because they contain lower levels of toxic metals and pollutants than conventional foods. The consumers' attitudes towards purchasing organic foods are mainly driven by the health benefits of these foods (Kaviani Darani et al. 2025). The results of some investigations demonstrate that health factors are among the most important factors that remarkably influence consumers' willingness to buy organic foods. Organic foods have a competitive advantage over conventional foods, which is due to the nutritional properties of organic foods (Gundala and Singh 2021; Jakubowska et al. 2024). The advantages of organic foods can be mentioned to higher antioxidant and antimicrobial agents in particular terpenoids, phenols, anthocyanins, and flavonoids in organic herbs, vegetables, fruits, and edible mushrooms, enhanced levels of omega‐3 fatty acids in organic dairy products and edible vegetables and fruits oils, and boosted fatty acid profiles in organic meat products (Kaviani Darani et al. 2025; Samani et al. 2021; Vigar et al. 2019).

## 3 Classification of Functional Foods

In the past decade, while organic and natural foods have become increasingly popular, functional foods have also attracted considerable public interest. Likely due to their potential to improve or maintain health, the food industry is keen to market these foods. However, such claims are usually supported by a lack of awareness among the public, which is often associated with poor dietary choices (Vignesh et al. 2024). The concept of functional foods has evolved considerably, with an emphasis on the role of diet in boosting health and wellness. For scientific studies, dietary planning, and a balanced diet, as well as consumer education, it is essential to classify these foods. The functional foods and beverages include a wide range of ingredients such as medicinal and aromatic plants, spices, legumes, oilseeds, cereals, fruits, vegetables, dairy products, beverages, algae, fish and seafood, and medicinal and edible mushrooms, which offer unique health‐promoting features (Essa et al. 2023; Ghasemi Pirbalouti et al. 2013, 2014; Vignesh et al. 2024). A close examination of their composition reveals a wide and diverse range of valuable biologically active substances that distinguish them from traditional and folkloric foods and beverages (Ghasemi Pirbalouti et al. 2014; Rezaei and Ghasemi Pirbalouti 2019). These compounds, ranging from a diverse group of vitamins, minerals, phytochemicals, essential fatty acids, and other helpful compounds, give these functional foods properties beyond basic nutrition. The functional foods and beverages, whether derived directly from natural sources or enriched with additional bioactive compounds, have a very unique composition of compounds that distinguishes them as agents of potent holistic health (Martirosyan et al. 2022; Singh, Pal, et al. 2023). Since there is a positive association between functional foods and health in achieving optimal health and reducing disease risk, understanding the functional mechanisms through which nutraceuticals (a combination of “nutrition” and “pharmaceutical” that refers to products that can be used to prevent or treat diseases) exert their effects is essential to understanding their transformative potential. Indeed, these consumables operate at the levels of cellular and molecular and modulate physiological functions in ways that go beyond the conventional comprehension of nutrition (Mittal et al. 2024).

Generally, there are three classifications of the functional foods including (Hasler and Brown 2009):

- Conventionally used foods are foods that have not undergone any modification, like pulses and grains, dairy, fish, vegetables, and edible mushrooms, fruits, etc., which are thought to have strong health benefits;
- Modified foods are foods that have been enriched or fortified with one or more specific nutrients to enhance health benefits. For instance, food products enriched with sterols and omega‐3 (n‐3) fatty acids and other bioactive substances, beverages fortified with calcium, antioxidants, and various vitamins, fortified bread with vegetables as a natural source of folates as well as some food products enriched with plant‐based natural products like herbs;
- Food ingredients.

### 3.1 Functional Foods With Prebiotics Origin

In recent years, prebiotics and probiotics have attracted much attention due to their ability to alter the gut microbiota and influence a number of factors associated with health and diseases. The prebiotics are indigestible fibers that specifically improve the growth and activity of beneficial bacteria. They are live microorganisms that, when consumed in sufficient amounts, provide health benefits and show promise in modifying the composition and function of the gut microbiota (Obayomi et al. 2024). The first to provide a specific definition of prebiotics were “Gibson” and “Roberfroid”. The definition they provided for prebiotics was that “prebiotics are considered to be food components that promote the growth and activity of one or a specific group of microorganisms inhabiting the gastrointestinal tract and can improve the health status of the host.” Inulin, as a group of naturally occurring polysaccharides and oligo‐fructose are the most common form of prebiotics, which selectively up‐regulate the beneficial gut microorganisms like bacteria and fungi (Blaak et al. 2020). Moreover, the probiotics are rapidly propagated using prebiotic compounds found in beneficial microorganisms such as bacteria and fungi, and/or certain plant‐derived compounds like oligosaccharides (Enteshari Najafabadi et al. 2024).

Sometimes in practice, a combination of probiotics and prebiotics is used, due to their synergistic action in functional foods. The symbiotic products can be useful to the consumer through the survival and establishment of selected live microorganisms in the digestive system (Ballini et al. 2023). Enteshari Najafabadi et al. (2024) reported that polysaccharide fractions (cyanidin‐3‐O‐β‐glucoside, N‐acetylcysteine, and glutamic acid) from some herbs from the Asteraceae family such as dandelion ( *Taraxacum officinale* F. H. Wigg.), chicory ( *Cichorium intybus* L.), and thistle‐like ( *Gundelia tournefortii* L.) enhanced the growth of *Lactobacillus rhamnosus* as a native probiotic in comparison to two commercial prebiotics, i.e., inulin and dextrose, so that they can be useful in producing functional foods and nutraceuticals. The best studied prebiotics, which are found in various foods including some herbs, vegetables, and fruits (garlic, leeks, onions, artichokes, asparagus, shallots, bananas, etc.), are inulin and fructo‐oligosaccharides or FOS [both types of β (1–2) fructans] (Di Renzo et al. 2019).

### 3.2 Functional Foods Derived From Animals and Containing Prebiotics

Foods of animal origin have a high potential to provide various important nutrients such as proteins, fats, iron, and vitamin B_12_. In addition, there are some bioactive components in foods of animal origin that ensure human health and fight some disorders. Generally, animal foods are very popular among society because they act as functional foods and actually provide additional health benefits. For instance, milk is one of the best and most important sources of calcium, and it also contains bioactive peptides and probiotics. Marine animals are also rich in valuable compounds such as omega‐3 fatty acids and chitin/chitosan, which have a special place in the food industry, particularly the functional foods. The meat and meat products can meet nutritional needs and improve health due to their high content of conjugated linoleic acid, bioactive peptides, and iron. Generally, the bioactive components, including organic or inorganic molecules such as carbohydrates (especially in honey), vitamins particularly fat‐soluble vitamins stored in the liver, fatty tissues, and muscles (fish, meat, egg, milk, and dairy products), fatty acids or lipid biomolecules (fish oil, salmon, tuna, meat, and cheese), and omega‐3 fatty acid (fish, seafood, dairy products), as well as minerals and various micronutrients and macronutrients in some valuable foods like milk and dairy products (cheese, yogurt, kefir, and crud), fish and seafood, meats, poultry, and eggs, are naturally available in functional foods derived from animal sources and positively affect human health (Dixit et al. 2023). Additionally, the bioactive peptides derived from animal proteins have antimicrobial effects that can be used as antibacterial agents in fortified products. These compounds are also considered natural antioxidants, thus highlighting the therapeutic potential of these compounds with significant health implications for humans (Cuchillo‐Hilario et al. 2024).

Results of previous investigation (Ma et al. 2022) have shown that consuming dairy products like yogurt with high probiotic concentrations can prevent the deterioration of periodontal status and act as a protective factor. The oral microbiome also plays a very important role in this regard; therefore, consumption of probiotic yogurt is recommended to prevent tooth loss. *Bifidobacterium infantis* , *B. longum* , *B. lactis* , *B. breve* , *Lactobacillus casei* , *L. acidophilus* , and *L. rhamnosus* are among the probiotic strains that are used in encapsulated form to add to yogurt (Burgain et al. 2011).

Demineralization and remineralization are two opposing stages that occur in teeth and affect their structure and strength. Demineralization is the loss of important minerals like calcium and phosphate, particularly from the enamel and other hard tissues. On the other hand, remineralization is the stage of restoring those lost minerals, often through the deposition of calcium and phosphate in particular from saliva. The most important factors that indicate the balance between the demineralization and remineralization stages of tooth enamel are the calcium concentration of saliva and dental plaque. These are considered essential indicators that should be considered in the control and treatment of dental diseases. The results of some studies have shown that the consumption of yogurt and milk can reduce the risk of dental caries, which is exerted through the high level of calcium in yogurt and milk. In the market, there is a wide range of probiotics products that come in various forms, including sucking tablets, chewing gums, lozenges, and dairy products, which contain different species of probiotic bacteria (Malavalli et al. 2022; Poureslami et al. 2013; Srivastava et al. 2016). The probiotics used for oral health should contain a variety of bacteria. The use of probiotics for the treatment of oral illnesses such as dental caries, periodontal diseases, and halitosis is very important and significant (Vuotto et al. 2014). Most naturally occurring probiotics can be found in fermented dairy products, such as curd, cheese, yogurt, and kefir, which are components of the human diet and are easily available (Jindal et al. 2012).

Due to the valuable components present in milk and dairy products, particularly some minerals and vitamins, these products have a prominent position in the functional foods sector. Milk contains a large amount of a variety of essential elements and biologically active substances like lactoferrin, immunoglobulins, lactoperoxidase, and oligosaccharides, which have beneficial impacts on oral and dental health (Bolat et al. 2024).

In the production of functional yogurt, various natural compounds are used as ingredients, such as fruits, pure fruit, seeds, peel, pulp, and flour (Akdeniz 2023; Shakerian et al. 2012; Turgut and Diler 2023; Yapa et al. 2023). In a previous study, banana pulp was used to enrich yogurt produced with *Levilactobacillus brevis* (Abdelazez et al. 2024).

### 3.3 Functional Food of Plant Origin

Antioxidants are non‐enzymatic defenses of the organism against reactive O, N‐species, which are essential for human health. Most antioxidant substances enter the organism through the diet (Bajalan et al. 2016; Rezaei and Ghasemi Pirbalouti 2019). Antioxidants have great importance because they can decrease oxidative stress causing damage to biological cells and molecules. Antioxidants play a key role in the treatment of many diseases by acting as free radical scavengers and decreasing the extent of oxidative damage (Bajalan et al. 2017). There are many indications that the toxicity and malnutrition effects of synthetic antioxidants are added to foods. Some medicinal and aromatic plants, vegetables, fruits, nuts, and spices contain a wide variety of free radical scavenging molecules that have antioxidant properties (Ghasemi Pirbalouti and Gholipour 2016; Ghasemi Pirbalouti et al. 2014). These plants and their phytochemicals, particularly terpenoids (especially phenols like thymol and carvacrol) and polyphenols (phenolic acids, flavonoids, anthocyanins, tannins, etc.), are plant‐based natural ingredients; most of these compounds, by having antioxidant properties, prevent oxidative stress (Farhadi et al. 2025; Rezaei and Ghasemi Pirbalouti 2019). For this reason, that's why the best approach to get enough antioxidant agents is to place nutrients and antioxidants in the diet, like various herbs, spices, vegetables, and fruits. Other classifications of functional foods include functional food of fruit origin, functional food of nut origin, and functional food of animal or livestock origin, some of the most important of which are mentioned in Table 1.

**TABLE 1:** Some of the most important organic and functional foods of plant and fruit, animal, probiotics, and nut origin.

Origin	Nutritional composition	Bioactive compounds	References
Tomatoes ( *Solanum lycopersicum* L.)	Vitamins, minerals (calcium, potassium, etc.), fiber, protein, essential amino acids, monounsaturated fatty acids, carotenoids, and phytosterols	Lycopene, α‐cryptoxanthin, carotene, β‐cryptoxanthin, β‐carotene, zeaxanthin, lutein, phytoene, phytofluene, neoxanthin, violaxanthin, cyclo‐lycopene‐neurosporene	(Perveen et al. 2015; Na et al. 2025)
Spinach ( *Spinacia oleracea* L.)	Minerals (iron, copper, phosphorus, zinc, selenium), vitamin B complex (niacin and folic acid), ascorbic acid, omega‐3‐fatty acids	Patuletin, spinacetin, spinatoside, jaceidin, flavone, flavonoid derivatives, and 20‐hydroxyecdysone. Flavonoids (spinacetin, patuletin, methylenedioxyflavone derivatives)	(Cerulli et al. 2024)
Bananas (*Musa* spp.)	Vitamins (Pantothenic acid (B5), Pyridoxine (B6), Vitamin C), Minerals (Magnesium, Phosphorus, Potassium, Sodium, Zinc), dietary fiber, pectin, and β‐carotene	Polyphenols, terpenoids, alkaloids, steroids, anthocyanins, tannins, and fatty acids, phenolic (trienoic acid, dienoic acid, vanillic acid, caffeic acid, ellagic acid, 13‐octadecanoic acid, palmitic acid, carvacrol, etc.)	(Mondal et al. 2021; Sidhu and Zafar 2018; Huang et al. 2021)
Apple (*Malus* spp. particularly *Malus domestica* )	Organic acids, fibers, minerals (potassium, magnesium, calcium, sodium, phosphorus, zinc, manganese, copper, iron), vitamins (A, B_1_, B_2_, B_3_, B_5_, B_6_, B_9_, C, E), and protein	Polyphenols (phenolic acids, dihydrochalcones), phenolic acids (chlorogenic acid, caffeic acid), dihydrochalcones (phloridzin, phloretin, phloretin‐2′‐xyloglucoside), flavonoids (epicatechins, proanthocyanidins), and anthocyanins	(Patocka et al. 2020; Arnold and Gramza‐Michalowsk 2024)
Blueberries ( *Vaccinium corymbosum* L.)	Dietary fibers, vitamins (C and K, and low levels of A, B, and E), and minerals (calcium, iron, magnesium, manganese, and zinc)	Polyphenols (anthocyanins and flavonols), phenolic acids, carotenoids (lutein), flavonols (kaempferol, quercetin, myricetin), flavanols (proanthocyanidins), and phenolic acids (hydroxycinnamic acids)	(Krishna et al. 2023)
Pomegranate ( *Punica granatum* L.)	Amino acids, minerals, vitamins C, calcium, iron, phosphorus, retinol, riboflavin, ferulic acid, carbohydrates, punic acid, heneicosenoic acid, nonadecanic acid, steriac acid, palmitic acid, oleic acid, linolenic acid, linoleic acid, and octoic acid	Ellagitannin, tannins, saponin, flavonoids, alkaloids, quinones, cardiac glycosides, terpenoids, phenols, coumarins, steroids, and anthocyanins (delphinidin, cyanidin, pelargonidin)	(Ghasemi Pirbalouti et al. 2010; Maphetu et al. 2022)
Licorice ( *Glycyrrhiza glabra* L.)	Proteins, amino acids, polysaccharides, simple sugars, mineral salts (calcium, phosphorus, sodium, potassium, iron, magnesium, silicon, selenium, manganese, zinc, and copper), pectins, resins, and vitamins (B_1_, B_2_, B_3_, B_5_, E, and C)	Alkaloids, cardiac glycosides, tannins, anthraquinones, saponins, flavonoids, and polysaccharides (glycyrrhizic acid, glycyrrhetinic acid, etc.), flavonoids (liquiritigenin, liquiritin, Licoflavonol, Isoliquiritigenin, Glabridin, Glycyrol, etc.), Triterpenoids (Glycyrrhetinic acid, Glycyrrhizic acid, etc.)	(Dang et al. 2024; Pastorino et al. 2018)
Almond [ *Prunus dulcis* (Mill.) D.A. Webb.]	Unsaturated fatty acids, proteins, low sugar content, minerals (potassium and phosphorus), amino acids (arginine), dietary fiber, phytosterols, and essential vitamins (especially α‐tocopherol)	Polyphenols, hydrolysable tannins (ellagitannins, gallotannins, and ellagic acid), proanthocyanidins and flavonoids, phenolic acids, lignans, stilbenes, and isoflavones	(Silva et al. 2025; Barreca et al. 2020)
Walnut (Persian or English) walnut ( *Juglans regia* L.)	High levels of unsaturated fatty acids, phenolic compounds, digestible proteins, and dietary fiber	Juglone, ellagic acid, tannic acid, tannic acid, 3‐methoxy derivative of juglone, and tetralones	(Jahanban‐Esfahlan et al. 2019)
Hazelnut ( *Corylus avellana* L.)	Folic acid (vitamin B_9_), antioxidants (tocopherols and polyphenols), mineral salt composition, calcium, magnesium, and potassium	Phenolic Acids (salicylic acid, 4‐hydroxysalicylic acid, gallic acid, vanillic acid), flavonoids (aglycone quercetin, myricetin), flavonoid glycosides, ellagic acid, and hydrolysable tannins (ellagic acid hexoside isomer, ellagic acid pentoside isomer)	(Bottone et al. 2019; Rondanelli et al. 2023)
Peanuts ( *Arachis hypogaea* L.)	Tocopherols, protein, amino acid (lysine, methionine, threonine), high amounts of vitamins B (thiamine, riboflavin, niacin, folate, and vitamin B_6_), and fiber, calcium, iron, potassium, unsaturated fatty acids, and linolenic acid	Phenolic compounds, stilbenes, lignans, isoflavonoids, *P*‐coumaric acid, Resveratrol, *P*‐hydroxybenzoic acid, Quercetin, Luteolin	(Çiftçi and Suna 2022)
Pistachios ( *Pistacia vera* L.)	Protein, dietary fiber, potassium, magnesium, vitamin K, γ‐tocopherol, mono‐ and poly‐unsaturated fatty acids (oleic and linoleic acids)	Lutein, anthocyanin, phytosterols, xanthophyll carotenoids, flavanones (quercetin, kaempferol, luteolin, apigenin), and isoflavones (genistein, daidzein)	(Mandalari et al. 2021)
Milk	Liposoluble (A, D, E), hydro‐soluble vitamins (B complex and vitamin C), caseins (major protein), and calcium	Medium chain triacylglycerols (TAGs), palmitic acid (C16:0), amino acids (cysteine), carotenoids, equol (a polyphenolic metabolite), lactoferrin, immunoglobulins, lactoperoxidase, and oligosaccharides	(Roy et al. 2020; Khan et al. 2019)
Eggs	Protein, amino acid, monounsaturated, and polyunsaturated fatty acids (n‐3) fatty acids, palmitic acid (C16:0), stearic acid (C18:0), oleic acid (C18:1 n‐9), docosahexaenoic acid DHA (C22:6 n‐3), vitamins (A, B, D, E, K), and calcium	Carotenoids (lutein, zeaxanthin) and antioxidant compounds	(Cuchillo‐Hilario et al. 2024)
Functional yogurt	Antioxidants, vitamins, minerals, and proteins	Probiotics, prebiotics, synbiotics (*Lactobacillus*, *Bifidobacterium* species, *Streptococcus salivarius* subsp., etc.)	(Sarıtaş et al. 2024)

Nuts are one of the most important sources of proteins and essential amino acids. The nutritional value of a protein determines the essential amino acid contents and their digestion capability. Although the proteins in nuts are known as incomplete proteins due to the lack of all types of the essential amino acids, they have some health benefits and are also used in enriching functional foods. Additionally, the nuts contain various amounts of beneficial macronutrients or major minerals [> 100 mg/day] and micronutrients or trace elements [< 100 mg/day] including calcium, magnesium, potassium, zinc, iron, etc. According to the results of previous studies, periodontitis is associated with a deficiency in serum micronutrients, which usually increases the disease risk (Van der Velden et al. 2011). Minerals act as cofactors in enzymes, so their presence is appropriate and balanced, needed in different body compartments for numerous physiological processes. In addition, the minerals have structural functions that are of particular importance to bones and teeth (Varela‐López et al. 2016). Other beneficial bioactive substances found in the nuts with known health benefits include tocopherols, vitamins, and phenolic compounds (Gonçalves et al. 2023; Hadidi et al. 2025).

### 3.4 Bioactive Compounds From Marine Sources

Marine invertebrates and algae contain valuable nutritional and important medicinal compounds, and natural compounds derived from them have made a significant contribution to contemporary medicine (Sigwart et al. 2021). The main and specialized bioactive compounds produced by marine biological resources are various natural organic compositions such as peptides, proteins, polyethers, fatty acids, polysaccharides, and enzymes. The marine resources have high potential as functional ingredients in food due to some important and distinctive features, such as proteins with high functional properties including antibacterial activity, foaming ability, and gel‐forming capacity (Khora 2013).

One of the most promising sources for the development and sustainable production of naturally occurring bioactive metabolites under environmentally friendly conditions is microalgae. These photosynthetic organisms live in aquatic environments and produce a certain number of fatty acids and carotenoids. Additionally, they are rich in bioactive constituents such as phenolic compounds, flavonoids, anthocyanins, etc., each of which has its own unique functional properties (Centella et al. 2017; Del Mondo et al. 2021). Marine‐based nutrients can be obtained from a variety of sources, including marine plants, sponges, and microorganisms. Each of these contains a unique set of biomolecules that allow them to thrive in their environment. Due to the high levels of polyphenols and carotenoids in seaweed, these compounds are now gaining attention in scientific research and are being investigated as excellent sources of antioxidants. Marine‐derived products, which also originate from post‐harvest processing of fish and seafood, are emerging as the most promising sources of marine food components. The main ingredients include shark liver oil, peptides, chitin and its derivatives (like chitosan), enzymes, seaweed, protein hydro‐lysates, and as well as fish oil that mainly contains omega‐3 polyunsaturated fatty acids, vitamins A, D, and E (Shahidi and Ambigaipalan 2015). In addition, some marine microorganisms have the potential to produce novel glucosidase inhibitors (Trang et al. 2021). Among the microorganisms that have the most nutritional applications and bioactive properties are *Cephalopods*, *Bivalvia*, and *Gastropoda* (the phylum Mollusca). Marine and estuarine mollusks have a diverse range of applications that are currently used as food and nutritional sources. For this reason, they have attracted the attention of food technologists and dieticians (Chakraborty and Joy 2020).

In fact, many bioactive substances from marine sources have been considered and utilized in many potential study areas in dental sciences due to their therapeutic and nutritional values. Results of investigation by Ibrahim et al. (2018) have indicated that some compounds derived from marine sources, like chitosan, have been used in bio‐adhesives, calcium hydroxyapatite, tissue regeneration gel, gypsum, and algal extracts in the field of dentistry. Additionally, they reported that the biologically active compounds from marine sources can have inhibitory impacts on *Streptococcus mutans* and which can be used to maintain dental health (Ibrahim et al. 2018).

Chitosan, as the major derivative of chitin, is the product of N‐deacetylated chitin. Chitosan is an important biopolymer in nature and the only positively charged (cationic) polysaccharide. The main sources of chitosan, as a natural biopolymer and macromolecule can include the exoskeletons of crustaceans like shrimp and crabs, as well as in the cell walls of fungi, insects, and some plants (Ghasemi Pirbalouti et al. 2017; Karimlar et al. 2025). Chitosan has many applications in sustainable agriculture (Rezaei‐Adl et al. 2025) as well as various industries such as food (processing, packaging, and preservation), biomedical, health care, pharmaceuticals, paper, biodiesel, and other uses. Chitosan gained significant interest for its safety, antifungal, antibacterial, biodegradability, biocompatibility, and antioxidant activities due to its rich amino and hydroxyl groups. Chitosan is a biodegradable, non‐antigenic, non‐toxic, and biocompatible natural polymer (Ghasemi Pirbalouti et al. 2017, Karimlar et al. 2025).

Generally, Huang et al. (2021) reported that the bioactive compounds and molecules from marine sources such as chitosan, bio‐adhesives, tissue regeneration gel, calcium hydroxyapatite, gypsum, algal extracts, etc., can be health benefits in dental caries, gingivitis, periodontitis, halitosis, and oral cancer. Moreover, these highly valuable molecules have potential use as functional food ingredients for oral health. For instance, alginate extracted from seaweed, as a non‐reducing sugar, can be added to food products in the industry as a substitute for sucrose to reduce the rate of dental caries (Deng et al. 2014). However, the results of a previous study (Onyango et al. 2020) indicated that alginate cannot completely prevent dental caries, and further investigation is needed in this field. Jun et al. (2018) stated that fucoidan, as a sulfated polysaccharide in the seaweed species ( *Fucus vesiculosus* F85), as a compound extracted from the marine sources, has shown significant antimicrobial effects against *Streptococcus mutans*; however, no biofilm removal was found by them. Likely, this discrepancy is due to different research conditions and methods, which require further investigation. Clinically, gingivitis plaque‐induced has various characteristics, and the most important factors in this regard are mainly predicted based on the presence or absence of bleeding on probing. As a result, Gingival Index (GI) and Bleeding on Probing (BOP) are among the main parameters to characterize gingivitis. In this context, the most important marine bioactive substances for relieving anti‐gingivitis reported by researchers include the extracts from sea cucumber and algae, as well as n‐3 polyunsaturated fatty acids (PUFAs). Chitosan and the sea cucumber extract have also been reported to be beneficial for periodontal health (Chapple et al. 2018).

It can be concluded that the use of natural compounds in the production of functional foods, in addition to affecting the physicochemical and sensory properties of these products and increasing marketability, can also improve the health of consumers. These enrichments with natural compounds and phytochemicals can also be carried out and investigated to improve oral and dental diseases.

## 4 Oral and Dental Diseases

Oral diseases are a major global public health problem that is highly prevalent and carries a heavy health and economic burden, affecting and severely reducing the quality of life of affected individuals (Peres et al. 2019). Excessive plaque accumulation, following poor oral hygiene, can lead to a range of diseases and conditions, including oral diseases such as caries and periodontal diseases (Omidsalar et al. 2022). In the oral cavity, more than 25 species of microorganisms have been identified, including known pathogens such as *Streptococcus mutans* , *Porphyromonas gingivalis* , and *Aggregatibacter actinomycetemcomitans* , which are clearly directly associated with caries and periodontal disease. Dental caries (tooth decay), periodontal disease, tooth loss, and cancers of the lips and oral cavity are among the most common and important oral diseases worldwide (Peres et al. 2019; Zhang et al. 2025).

### 4.1 HPV and Oral Squamous Cell Papilloma

Human papillomavirus (HPV) can infect various parts of the body, including the mouth, and cause warts. Oral squamous papilloma, known as a common benign epithelial neoplasm of the oral cavity, is a painless lesion that can usually occur at any intraoral site, but is most commonly found on the lips, tongue, cheek mucosa, hard, and soft palate. Most papillomas are single. This wart‐like lesion has numerous projections and tends to be pedunculated. The papillary projections may be pointed and finger‐like or rounded and cauliflower‐like, which the resulting lesion might be white, raspberry‐like, or pink in color. If there is excessive keratinization (cornification), the lesion appears white, and if the lesions are less keratinized, they are often raspberry‐like or pink. Squamous papillomas are usually single and less than 1 cm in size. They are often clinically indistinguishable from common warts (verrucae vulgaris), which are a common lesion found on the skin and sometimes on keratinized regions of the oral mucosa, such as the vermilion aspect of the lips, hard palate, and gingivae (Betz 2019; Prabhu and Wilson 2013).

### 4.2 Oral Candidiasis

Oral candidiasis, also commonly known as oral thrush, is an infection caused by yeast (a type of fungus) called *Candida*. Oral candidiasis is the most common opportunistic infection caused primarily by overgrowth of *Candida albicans* in the oral cavity. It is also increasingly associated with non‐albicans *Candida* spp., especially in patients who have had a history of multiple azole (heterocyclic compounds) antifungal treatments. Oral candidiasis is divided into primary and secondary infections, with primary infections limited to the mouth and its surrounding area, while secondary infections are associated with systemic mucocutaneous manifestations. The disease is typically classified into acute and chronic types, including pseudomembranous, erythematous, and hyperplastic variants as well as other *Candida*‐associated diseases such as denture stomatitis, angular cheilitis, and median rhomboid glossitis. The acute form of *Pseudomembranous candidiasis* (oral thrush) is classified as primary oral candidiasis and is known as a classic candidal infection. Infection is often seen in people taking antibiotics and immunosuppressive medications or those with a disease that weakens the immune system (Tamai and Kiyoura 2025; Vila et al. 2020).

### 4.3 Root Canal Infection

Root canal infection is an important infection of the inner core of a tooth that affects the root. It is usually caused by a variety of factors, including caries, gum disease, or trauma. In this regard, the resulting infection can cause severe pain and discomfort, and if left untreated, might lead to tooth loss. The root canal infections are caused by bacteria that invade the dental pulp and colonize the root canal system. The inflammatory response following severe infection is driven by bacterial populations as well as metabolic byproducts that enter the periradicular tissues through apical or lateral foramina. These reactions, in turn, can lead to lysis of both periradicular tissues and soft tissues as a result of osteoclasts recruitment and proteolytic activity of immune cells, including mast cells, neutrophils, and macrophages. Ultimately, this results in different proportions of immune cells, cellular debris, cholesterol crystals, osteoclasts, fibroblasts, and others, depending on the severity of the inflammation. There are several potential routes for pathogens to enter the root canal system, with dental caries being one of the most common. Infection includes fractures, trauma, exposed dentinal tubules, and iatrogenic causes, among other common sources of infection (Gulabivala and Ng 2023; Sakko et al. 2016; Wong et al. 2021).

### 4.4 Oral Cancers

Oral cancer is considered a malignant neoplasia that arises on the lip or oral cavity and is defined as a squamous cell carcinoma (SCC) because histologically, 90% of cancers in the dental area originate in squamous cells (Rivera 2015). The SCC is the most common type of cancer in the mouth. Several factors, including tobacco use and alcohol usage, have been identified as major risk factors for these oral malignancies. The main factor in the sharp increase in the incidence of oral and dental cancer is human papillomavirus infection (Rodríguez‐Molinero et al. 2021).

### 4.5 Periodontal Diseases

Periodontal disease is a chronic, non‐communicable inflammatory disease (NCD) that can affect the integrity of tooth‐supporting tissues and occurs when there is an imbalance between the periodontal microbiome and the host inflammatory response. Severe periodontitis affects at least 10% of the adult population and is the sixth most common disease worldwide. It causes irreversible damage to tooth‐supporting tissues, which has a significant negative impact on oral health. Over the past 30 years, evidence has shown that there is a link between oral diseases, including periodontitis, and a variety of systemic diseases. Periodontitis negatively affects systemic health through various biologically plausible mechanisms, while its successful treatment can reduce the risk of comorbidities (Chatzopoulos et al. 2024).

### 4.6 Dental Caries

Oral diseases, particularly dental caries, are one of the biggest health problems worldwide, affecting people of all ages. Oral diseases can cause pain, discomfort, disability, and sometimes death. It is estimated that approximately 3.5 billion people are affected by oral diseases. Tooth decay can develop over time, with a variety of factors contributing to its development. However, the major factor in the development of dental caries is related to dietary habits, which affect the natural flora of the oral cavity and occur together with host susceptibility. In addition to causing tooth loss, decay can also cause conditions such as mastication and swallowing difficulties, pain, and secondary complications such as pulpitis, infection, and osteomyelitis of the alveolar bone. Some of the most important bacterial species involved in dental caries include *Streptococcus sanguinis* , *Streptococcus mutans* , *Veillonella* spp., *Actinomyces* spp., *Bifidobacterium* spp., and *Lactobacillus fermentum* . Results of some studies have also shown that *Actinomyces gerencseriae* and other *Actinomyces* spp. are part of the normal flora of the oral cavity and respiratory tract and play an important role in the initiation of caries (Aas et al. 2008; Geleto et al. 2022; Xu et al. 2024). Results of investigations indicate that orthodontic patients with a high risk of caries before treatment tend to have higher levels of *S. mutans* in their saliva during treatment (Mirmohammadi et al. 2024). This is attributed to the rapid change in oral flora in favor of pathogenic microorganisms after the start of treatment with fixed appliances and the decrease in salivary pH 3 months after treatment, which contributes to the acidity of the oral environment and the appearance of white lesions.

### 4.7 Tooth Loss

In general, the main cause of tooth loss in people up to 50 years is dental caries and its sequelae. Actually, caries of 42% of teeth extractions, while the major cause of tooth loss in middle‐aged and elderly people is periodontitis, which accounts for 28% of the extractions (Virtanen et al. 2014). Early tooth loss can be caused by dental trauma, neonatal tooth extraction, early childhood caries, or periodontal problems, or might be due to the manifestation of systemic diseases. Caries and trauma are among the most common causes of early teeth loss (Spodzieja and Olczak‐Kowalczyk 2022).

### 4.8 The Oral and Dental Diseases Related to Oxidative Stress

Reactive oxygen species (ROS) and reactive nitrogen species (RNS) are reactive molecules that are often referred to as free‐radical species. In biological systems, oxygen free radicals (OFR) are among the most important radicals produced. The step‐by‐step transfer of electrons to molecular oxygen (O_2_) leads to the production of superoxide anions (O_2_•–), hydrogen peroxide (H_2_O_2_), and ultimately (•OH). Internal sources such as mitochondria, flavins (the flavin group is capable of undergoing oxidation–reduction reactions), adrenaline, dopamine, peroxisomes, neutrophils, macrophages, eosinophils, oxidase enzymes, peroxidases, and cytochrome P450 (P450s or CYPs) complexes, nicotinamide adenine dinucleotide (NADH), and external sources such as radiation, light rays, harmful chemical compounds in alcohol, cigarette smoke, etc., contribute to the generation of OFR. Nitric oxide (NO), as a species of reactive nitrogen radicals, is formed from the oxidation of L‐arginine to L‐citrulline by the enzyme nitric oxide synthase dependent on nicotinamide adenine dinucleotide phosphate (NADPH), which has a high diffusive power and also acts as a second messenger. Nitric oxide, once produced, reacts rapidly with the superoxide radical (O_2_^−^) and produces the powerful radical nitrite (ONOO‐) and hydroxyl (Abdelazez et al. 2024; Jomova et al. 2023; Mandal et al. 2022; Oyovwi and Atere 2024). ROS and RNS are naturally occurring and essential for life, while they can simultaneously lead to harmful oxidative stress and associated pathophysiology. Normal cells can protect themselves from oxidative stress by utilizing enzymatic antioxidants including superoxide dismutase (SOD), glutathione peroxidase (GPX), myeloperoxidase (MPO), and catalase and non‐enzymatic antioxidants including phenols, vitamins, minerals, and thiols (Demirci‐Çekiç et al. 2022; Kesarwala et al. 2016; Kozlov et al. 2024).

Oxidative stress (OS) is caused by an imbalance in the body's redox state, during which free radicals in the body increase and lead to damage to DNA (e.g., oxidative damage, hydrolytic damage, DNA strand breaks, and etc.), proteins, and lipids. Free radicals are produced through various metabolic pathways, such as aerobic metabolism in the mitochondrial respiratory chain, and play a key role in the onset and progression of various illnesses such as oral and dental diseases (Chaudhary et al. 2023; Guan et al. 2022). Importantly, abnormally increased levels of ROS due to oxidative stress (OS) can lead to cell death, decrease the osteogenic/odontogenic capacity of DPSCs (dental pulp stem cells), and subsequently exacerbate the inflammatory state. Therefore, it has been suggested that OS can play a critical role in uncontrolled inflammation and delayed healing in various diseases. Some investigators have reported in their results that “ROS” and “OS” markers, myeloperoxidase (MPO), and 8‐isoprostane [is a non‐enzymatic peroxidation product of arachidonic acid (AA)], are increased in pulp tissues (Dogan Buzoglu et al. 2023; Ibrahim et al. 2020; Li et al. 2024). Excessive distribution of ROS can lead to a number of detrimental consequences, such as tissue deterioration and an antagonistic cycle between the immune‐inflammatory cascade and ROS. Subgingival dental plaque triggers contain pathogenic bacteria and lipopolysaccharides (LPS), which stimulate TNF‐alpha and other Toll‐like receptors through their DNA. Inflammatory cytokines lead to the release of ROS from hyperresponsive PMNs (polymorphonuclear leucocytes). On the other hand, osteoclasts are also activated by the activation of toll‐1, the canonical Toll receptor (Toll‐1) [is activated by the cytokine Spatzle (Spz‐1)], and nuclear factor (NF)‐κB proteins, increasing the concentration of MMPs (matrix metalloproteinases), which both of which ultimately lead to tissue destruction. During periodontal tissue destruction, oxidized proteins, inflammatory mediators, and lipid peroxides are produced in large quantities (Patil et al. 2024; Wadhawan et al. 2020). Oxidative stress is implicated in the development of periodontitis (a chronic inflammatory disease of the periodontal tissue), which is caused by dysregulation of regulation of the host inflammation in response to bacterial infection. The results of previous studies have shown that serum total antioxidant levels (TAC) and salivary capacity are lower in chronic periodontitis compared to controls (Ahmadi‐Motamayel et al. 2017; Kumar et al. 2017). It has also been shown that 8‐isoprostane and malondialdehyde (MDA), as biomarkers of lipid peroxidation (a pathway mediated by oxidative stress), are increased in patients with chronic periodontitis (Akalιn et al. 2007; Pradeep et al. 2013).

Oxidative stress is believed to play a role in oral diseases such as lichen planus (LIE‐kun PLAY‐nus) and oral cancer. The presence and persistence of inflammation cause the release of free radicals through various mechanisms, including DNA damage, lipid peroxidation, protein damage, oxidation of antiproteases, and release of pro‐inflammatory cytokines, leading to damage caused by free radicals. The detrimental role of free radicals in carcinogenesis has been studied and tested for decades. Free radicals facilitate the development of oral cancer through the changes they cause to DNA (such as mutations, DNA‐mediated oxidation, mutation of tumor suppressor genes, and oxidative protein damage). Also, in oral lichen planus, the inflammatory infiltrate contains CD4 T lymphocytes (CD4+ cells) and serves as a source of reactive oxygen species that cause cellular damage (Ionescu et al. 2024; Subramanian et al. 2020).

Dental caries is known to be a multifactorial inflammatory disease, while the main factor in its development is usually the acidic by‐products formed during bacterial fermentation of carbohydrates. It is believed that the concept of an inflammatory response in dentin can be closely related to oxidative stress, which ultimately leads to the destruction of dental hard tissues. Based on the results of research, it has been established that the sensitivity of teeth to bacterial acids is due to the inhibition of dentinal fluid movement by high levels of sucrose, which consequently reduces the regulation of parotid hormone signaling from the hypothalamic (Spatafora et al. 2024; Tóthová et al. 2015).

The variety of oral ulcers is very large and complex. Some of these oral ulcers are associated with local stimuli. These local stimuli include mechanical (sharp edges of the root or crown remnants, etc.), physical (thermal burns, etc.), or chemical (strong acids or alkali, etc.). However, most oral ulcers occur due to a combination of local and systemic causes. One of the most common diseases of the oral mucosa in many parts of the world is recurrent aphthous stomatitis (RAS). This painful condition is characterized by the recurrent development of aphthous ulcers on the non‐keratinized mucous membranes of the mouth. Based on the results of some studies, it seems that the inflammatory reactions caused in recurrent aphthous stomatitis (RAS) are due to increased oxidative stress and an oxidant/antioxidant imbalance in the body (Estornut et al. 2024; Kurku and Yavuz 2022; Zeng et al. 2022).

## 5 Antioxidant Activity of Functional and Organic Foods in Oral and Dental Health

As research on functional foods and nutrients expands and their role in preventing and treating oral and dental diseases, it is imperative to carefully examine the strong scientific evidence supporting their claimed health benefits. Antioxidant compounds, which are abundant in many functional foods, can neutralize harmful free radicals, thereby reducing oxidative stress and inflammation in tissues. The plants and their bioactive compounds are considered a rich source of natural antioxidants and protect against the toxic effects of oxidants (Farhadi et al. 2025). The natural antioxidants are widely distributed in fruits and vegetables, medicinal plants, spices, probiotics, etc. (de Oliveira Zigmundo et al. 2022; Ghasemi Pirbalouti et al. 2019). Some natural antioxidants can be obtained through food. Higher intake of fresh fruits and vegetables has been associated with lower rates of oral cancer. The most researched compounds include curcumin, resveratrol, and green tea, which have anti‐inflammatory, antioxidant, immunomodulatory, and anticancer properties (Koh et al. 2011; Malcangi et al. 2023). Green tea has been shown to have a positive effect as a natural compound in the prevention and treatment of oral cavity tumors, particularly squamous cell tumors. Green tea modulates gene expression in oral tumors and significantly reduces the activity of various enzymes, including the activation of phase (I) aryl hydrocarbon hydroxylase (AHH) enzyme, cytochromes P450 (P450s or CYPs), cytochrome P450 reductases (CPRs), DT‐diaphorase (DTD), cytochrome b5 (CB5), cytochrome‐b5 reductase (CYB5R), and ultimately leads to an increase in phase (II) enzymes glutathione S‐transferases (GSTs) and UDP‐glucuronyl transferase (UDP‐GT) (de Oliveira Zigmundo et al. 2022; Srinivasan et al. 2008). The pulp of the fruit papaya ( *Carica papaya* ), which is rich in antioxidant nutrients, whose beneficial effects in the oral cavity and was able to inhibit *Streptococcus mutans* (Cespedes et al. 2021).

## 6 Bioactive Substances in Functional and Organic Foods

Natural foods and beverages with antimicrobial properties, very significant potential in managing halitosis or bad breath, through reducing the burden of volatile sulfur compound (VSC)‐producing bacteria in the oral cavity (Hamrun et al. 2023; Pellerin et al. 2021a, 2021b). Some plant‐based foods and natural beverages contain essential nutrients and biologically active substances such as phenols, flavonoids, anthocyanins, tannins, etc., which have inhibitory effects on bacterial growth and metabolism (Bae et al. 2022; Czerkas et al. 2024; Ghasemi Pirbalouti et al. 2014; Shamsudin et al. 2022). For instance, a group of natural antioxidants, including catechins, particularly epigallocatechin gallate (EGCG), which is abundant in green tea ( *Camellia sinensis* ), has been shown to target key bacterial species involved in VSC production like *Fusobacterium nucleatum* and *Porphyromonas gingivalis* (Hengge 2019; Higuchi et al. 2024; Kong et al. 2022). Catechins in green tea reduce the formation of odorous sulfur compounds by interfering with the enzymatic activity of bacteria and also improve oral health. The bactericidal effect of catechins can be attributed to their production of hydrogen peroxide (H_2_O_2_). EGCG can disrupt biofilms by damaging bacterial membranes and degrading exopolysaccharides (EPS) (Gopal et al. 2016; Kong et al. 2022).

Cranberry juice, as a beverage with antimicrobial and antioxidant properties has received much attention due to its active compounds in inhibiting pathogenic bacteria. The high content of polyphenols, including A‐type (A) linkage proanthocyanidins (PAC), in American cranberry ( *Vaccinium macrocarpon* ) shows great potential for the prevention of oral diseases (Feghali et al. 2012; Pappas and Schaich 2009). The PACs in cranberries can disrupt the adhesion of bacteria to oral surfaces (tongue and teeth). This interference with biofilm formation can potentially limit the ability of anaerobic bacteria to colonize the oral cavity and metabolize proteins into volatile sulfur compounds (VSCs). Some investigators have reported a reduction in bacterial counts and VSC levels in people who consumed cranberry‐containing products, suggesting their potential use as a dietary intervention for halitosis (Castellanos et al. 2024; Pellerin et al. 2021a, 2021b). Moreover, cranberry by‐products and phenolic extracts reduce bacterial adhesion to inorganic surfaces and epithelial cells (Rajeshwari et al. 2017). They can interfere with bacterial co‐aggregation and biofilm formation by pathogens involved in periodontal (gum) disease (Polak et al. 2013; Rajeshwari et al. 2017). Cranberry polyphenols are also immunomodulatory compounds that can reduce the production of pro‐inflammatory cytokines such as Interleukin‐1 beta (IL‐1β), Interleukin 6 (IL‐6), and tumor necrosis factor alpha (TNF‐α), as well as matrix metalloproteinases (MMPs) by mucosal cells (Bodet et al. 2007b; Tipton et al. 2014). Therefore, the unique chemical composition of this fruit, including the presence of A‐type (A) linkage proanthocyanidins (PAC), makes it a potential ingredient for functional foods. As has been observed in other previous studies, cranberry compounds can inhibit the activity of *Streptococcus sobrinus* and *Streptococcus mutans* (Babu et al. 2012; Feng et al. 2013; Philip et al. 2019). It has also been suggested that cranberry compounds can modulate the host immune response during periodontitis (Bodet et al. 2007a; Tipton et al. 2013).

Among natural foods, some medicinal herbs, spices, fruits, and raw vegetables exhibit significant antimicrobial activities (Ghasemi Pirbalouti et al. 2016). These natural foods can prevent the development of oral and dental infections due to their antibacterial effects. So that the fresh vegetables and herbs in the daily diet can have health benefits for oral and dental health (Anwar et al. 2025). It has been suggested that ending each meal with a hard food or fruit can be an effective way to prevent oral diseases such as tooth decay and periodontal disease. Apples ( *Malus domestica* ) are among the fruits commonly recommended as a means of cleaning teeth after meals, as they can stimulate the flow of alkaline saliva and neutralize the acids produced in dental plaque after carbohydrate consumption; as a result, apples have been included in health‐education programs (Rubido et al. 2018). Lycopene is a natural pigment synthesized by plants. Although it is found in fruits such as grapefruits and watermelons, the main source of lycopene seems to be ripe and fresh tomatoes ( *Solanum lycopersicum* L.). This compound is rich in antioxidant properties, which have been shown to have a positive influence in the prevention and treatment of chronic diseases. Therefore, it could also be useful in the treatment of potentially malignant oral diseases. Additionally, lycopene may act as a protective factor against oral cancer due to the regulation of lipid peroxidation and reduced glutathione (GSH) (Rodríguez‐Molinero et al. 2021).

Saponins and phenols in garlic ( *Allium sativum* L.) have high antioxidant properties. the ethyl linoleate compound in garlic acts as a potent anti‐inflammatory agent by reducing nitric oxide, interleukin (IL)‐1, TNF‐α, and prostaglandin E2 (PGE2). Flavonoids in garlic also have certain antiviral properties. Alliin (S‐allyl cysteine sulfoxide) is converted to allicin by an enzyme called alliinase. Allicin and other compounds of organosulfur in the bulbs (and leaves or stems) of the bulbous plants such as garlic, leek ( *Allium ampeloprasum* ), Iranian onion (*Allium jesdianum* Boiss. & Buhse.), moseer or Iranian shallot (*Allium hirtifolium* L.), etc. widely utilized in functional foods like dairy products and have antibacterial and antioxidant properties (Ghasemi Pirbalouti 2019; Ghasemi Pirbalouti et al. 2015). These components may have health benefits for the mouth and teeth. Since garlic is a source of prebiotic fiber, it is beneficial for dental health and proper digestion (Shooriabi 2021). Additionally, the bulbous herbs are rich sources of antioxidants and valuable substances such as vitamins A, B, C, and D, β‐carotene, and minerals like potassium, phosphorus, calcium, sodium, magnesium, iron, copper, and manganese, that in general, these substances can be useful for promoting oral and dental health (Aleebrahim‐Dehkordy et al. 2016).

Products such as propolis, grapes, and eucalyptus, which contain polyphenolic compounds, have shown activity against oral biofilm. It has also been found that eucalyptus extract is effective against halitosis (Kuang et al. 2018; Suzuki et al. 2018). Propolis, as a resinous compound made by honeybees from the buds of trees and other plant sources, has long been used in folk medicine as an antibacterial and antiviral agent for the treatment of inflammation. It has also been used for wound healing and as a local anesthetic, and its beneficial effects have been recommended for the treatment of purulent disorders (Hossain et al. 2022). Among flavonoid compounds, quercetin and chrysin are most widely found and distributed in the propolis of various honey bee species. These flavonoids are among the active compounds in plant resins, and their various biological activities, including the antimicrobial and anti‐inflammatory activities, have been proven (Farhadi et al. 2020; Zullkiflee et al. 2022). Some medicinal herbs, such as star anise fruit ( *Illicium verum* Hook. F.), clove ( *Syzygium aromaticum* L. Myrtaceae), cinnamon ( *Cinnamomum aromaticum* ), and ma‐kwean (*Zanthoxylum limonella*) have also been used since ancient times to maintain dental health (Atisakul and Saewan 2023). The essential oil of *Syzygium aromaticum* , which is obtained from the dried flower buds of the clove tree, has been utilized in clinical dentistry for centuries. Its main constituents include caryophyllene, eugenol acetate, and eugenol, which provide a unique feeling of cleanliness and freshness. It has also been shown to have antibacterial effects in oral infections caused by *Streptococcus mutans* (Pulikottil and Nath 2015; Yoo and Jwa 2018). In Thai folk medicine, *Zanthoxylum limonella* oil is used to prevent tooth decay and halitosis (Supabphol and Tangjitjareonkun 2014). *Zanthoxylum limonella* can inhibit the caries‐causing bacteria ( *Streptococcus mutans* ) in a dose‐dependent manner. Three main components of the essential oil of this plant are limonene, sabinene, and terpinen‐4‐ol, with sabinene being the main inhibitor of *Streptococcus mutans* growth (Park et al. 2019). Betel plant ( *Piper betle* L.) is also one of the plants with antimicrobial properties, which have been attributed to bioactive compounds, including chavicol, chavibetol, ally pyrocatechol, chavibetol acetate, ally pyrocatechol, ally pyrocatechol diacetate, etc., and hence it is widely used in the food industry (Roy et al. 2020). The antimicrobial activity of the betel leaves against various bacteria, including oral bacteria, has been proven, and bioactive molecules, sterols, have also been reported to be responsible for this antimicrobial activity (Heliawati et al. 2022; Singh, Singh, et al. 2023).

The role of probiotics in oral health is supported by years of research, although the number of human clinical trials conducted in this area is limited; yet, many patients are unaware of the potential benefits that dental probiotics can offer. The presence of beneficial microbiota, or so‐called probiotics, in the human body provides numerous benefits to humans, from preventing halitosis and cavities to improving gum health etc. (Beattie 2024; Eslami et al. 2019; Karbalaei, Keikha, Yousefi, et al. 2021). The good bacteria in oral probiotics can form biofilms to replace undesirable bacteria. These new biofilms not only support healthy teeth and gums and reduce inflammation, but they can also prevent bad bacteria from reaching the tooth enamel or gum tissue, where they cause problems. Therefore, fortifying various food products with probiotic strains such as milk, yogurt, kefir, yogurt drink, curd, butter, and cheese can produce substances such as vitamins B_6_ and B_12_, riboflavin (also known as vitamin B_2_), niacin (also known as vitamin B_3_), folic acid, and short‐chain fatty acids (SCFAs) like lactic acid and propionic acid. These food ingredients, collectively, can improve digestive function or even halitosis (Ghasemian et al. 2018; Giordani et al. 2021). Various genera of bacteria can be used as probiotics; however, the genera most commonly used as probiotic products include *Bifidobacterium* and *Lactobacillus* ( *B. bifidum* , *B. longum* , *B. infantis* , and *L. reuteri* , *L. rhamnosus* , *L. acidophilus* , *L. gasseri* , *L. casei* , *L. johnsonii* ) (da Silva et al. 2024; Vlasova et al. 2016). When taken orally, these bacteria can be used as cariogenic probiotics in the prevention of dental caries. It has also been reported based on the results of studies that probiotics significantly reduce the number of *Streptococcus sobrinus* and *S. mutans,* as two of the main causes of dental caries. The results of studies have shown that oral administration of probiotic lactobacilli effectively reduces physiological halitosis and also improves bleeding when probing periodontal pockets (Iwamoto et al. 2010). Hence, probiotic bacteria can be used as preventive bacteria in various dairy products (Luo et al. 2024; Patil et al. 2021). Milk contains most of the nutrients required for the growth and development of the human body, making it a highly nutritious and complete diet. Products made from fermented milk contain lactic acid bacteria, which have been linked to several positive effects on human health. Since natural and organic compounds, including phenols and polyphenols, are water‐soluble, these compounds can easily be enriched in milk to maintain oral health (Patange et al. 2023). On the other hand, oral probiotics are most effective when administered directly to the mouth via sublingual tablets, chewable tablets, milk, or probiotic drinks. However, there is a need to investigate the various aspects of products fortified with natural and organic compounds and provide sufficient evidence for their use (Lima‐Engelmann and Schneider 2022; Mishra et al. 2020; Stamatova and Meurman 2009).

Although bacterial infection is not responsible for halitosis in the early stages of periodontal disease, if periodontal disease persists and is not treated, persistent infections can affect oral conditions, leading to halitosis (Gomes et al. 2016; Yousefi et al. 2019). Pro‐inflammatory cytokines such as TNF‐α and IL‐1β play a pivotal role in periodontal disease. The researchers' results demonstrated that consumption of *Lactobacillus reuteri* ‐containing chewing gum as a probiotic product was able to significantly reduce the pro‐inflammatory TNF‐α and IL‐1β in gingival crevicular fluid with moderate levels of gingivitis inflammation and bacterial plaque. The potential therapeutic and protective effects of *L. reuteri* in periodontal diseases could be due to the presence of two potent bacteriocins, “reutericyclin” and “reuterin”, which can inhibit the growth of a wide range of pathogenic bacteria; the second possible impact could be related to the strong ability of *L. reuteri* to adhere to host tissues and compete with pathogens. In addition, considering the previously known anti‐inflammatory influences of this bacterium as an immunomodulator in gastrointestinal (GI) infections, it can be stated that *L. reuteri* is a viable therapeutic option with beneficial effects in periodontal diseases (Mahdizade Ari et al. 2024).

Janiani and Ravindran (2022) found that salivary levels of *Streptococcus mutans* were reduced in children with short‐term consumption of probiotic milk, reporting that although long‐term effects were not evident, probiotic milk was able to lead to significant reductions in this bacterium. The utilization of probiotic‐enriched cheese was also investigated in a previous study (Sarmento et al. 2019), and its potential to influence the oral microbiota in children, and positive results were reported for the use of this product. Babina et al. (2023) concluded in their research results that daily probiotic consumption led to a significant reduction in the level of *Streptococcus mutans* in the saliva of children. Parents' knowledge of child nutrition and their awareness of the interaction between unhealthy sugars in their child's diet and caries formation. Some of the scientific evidence on the use of functional and organic foods in oral and dental health, based on the results of clinical trial studies, is presented in Table 2.

**TABLE 2:** Functional products that promote oral and dental health.

Diseases	Product form		Source	Outcome	References
Infected pulp of primary teeth	Gingerols extract (mixture with calcium hydroxide)		Fresh ginger	In the treatment of infected root canals, ginger can be considered as a suitable and promising material as an obturation material or as an intracanal medicament for infected root canals. In general, the use of gingerols in endodontic materials could be a good opportunity for success	(Qasem et al. 2024)
Pulpectomy	*Aloe vera* gel (mixture with zinc oxide powder)		*Aloe vera* L.	The use of a mixture of zinc oxide powder and *aloe vera* gel is a suitable option in endodontic treatment in primary teeth, based on which clinical and radiographic results have been good and positive. Conducting a more detailed observational study with longer follow‐up could highlight the significant benefits of *aloe vera* in primary teeth as an obturating medium	(Khairwa et al. 2014)
Dental plaque	Chewing an apple		Apple	The results showed that chewing apples can have a positive mechanical effect on plaque removal. The greatest reduction in dental plaque was observed in the mesiobuccally and the least in the mid‐buccal level. However, based on the results, it was determined that chewing a yellow apple can be a more effective method in reducing dental plaque than chewing a red apple or using a toothbrush	(Mojarad et al. 2021)
Xerostomia	Thyme honey Oral rinses (20 mL of thyme honey diluted in 100 mL of purified water)		*Thymus* L.	Based on the results of this study, it has been determined that thyme honey has acceptable efficacy and safety in managing xerostomia. It has also been determined that this type of honey has been able to have a positive effect on other symptoms, including pain and taste loss, dysphagia (difficulty swallowing)	(Charalambous et al. 2017)
	*Aloe vera* mouthwash (50% *aloe vera* )		*Aloe vera* L.	The use of *Aloe vera* has had significant positive effects in reducing the symptoms of dry mouth. This plant has been able to reduce symptoms such as the need to drink water to swallow dry foods, decreased salivation, and mouth dryness upon waking, dry mouth during travel, and a burning sensation in the mouth	(Badooei et al. 2021)
	Mouthwash		*Plantago ovata* Forssk.	The results showed that *Plantago ovata* Forssk was able to effectively reduce oral mucositis, pain, and xerostomia compared to placebo (*p* < 0.05), and led to improved quality of life. The oral care protocol also reduced oral mucositis	(Hasheminasab et al. 2020)
Periodontal diseases	Lycopene (4 mg lycopene/day) for 2 weeks with oral prophylaxis [full mouth scaling and root planing (SRP) within 2 weeks]		Tomato	The results suggest that the use of lycopene as a supplement is a promising treatment for patients with moderate periodontal disease. It appears that the potent antioxidant compounds of lycopene can modulate the production of free radicals to inhibit tissue damage. Therefore, treatment with antioxidants, such as lycopene, can block the production of free ROS, and its effects may be therapeutically valuable and acceptable	(Belludi et al. 2014)
	Catechin		Green tea	The results demonstrated that in patients with periodontal disease, daily consumption of green tea along with scaling and root planning (SRP) treatment could significantly reduce the levels of the important inflammatory cytokine interleukin‐1 beta (IL‐1β) in saliva samples. As a result, it seems that a simple change in daily diet can help reduce inflammation and improve periodontal conditions	(Rezvani et al. 2022)
	Plant foods		Fruit + starch‐free vegetables	The results showed that in the elderly Korean population, there was an inverse relationship between the consumption of plant‐based foods and the prevalence of periodontal disease, and the prevalence of this disease decreased significantly with increasing consumption of plant‐based foods. According to the results, it can be stated that the consumption of plant‐based foods, including fruits and non‐starchy vegetables, can be included in the diet of the elderly in order to prevent periodontal disease. It is also suggested that further research is needed to better understand the mechanism of the effects of plant‐based foods on periodontal disease	(Kwon and Kim 2022)
Oral mucositis	Honey	Oral care protocol using honey (topically and mouthwash)	Indonesian natural and standardized flower honey	The results of this study showed that honey has protective and therapeutic effects on chemotherapy‐induced mucositis in children with cancer. Also, it was concluded that honey could be a suitable option as an oral care intervention for chemotherapy‐induced mucositis	(Nurhidayah et al. 2024)
		Topical	Local Saudi honey	The results indicated that a significant reduction in oral mucositis and aerobic pathogenic bacterial infections was observed in the group using honey for treatment	(Al Jaouni et al. 2017)
	Vegetable‐ fruit	Polyphenols, carotenoids, and lycopene	Vegetable and fruit juice	Based on the results of this study, it was found that the use of vegetable and fruit juices rich in phytochemicals was able to significantly reduce oral mucositis caused by combined endostar with concurrent chemoradiotherapy (CCRT) grade ≥ 2. It seems that the use of fresh vegetable juices due to their abundant phytochemicals can be a healthy, safe, and effective dietary strategy to reduce oral mucositis and its complications in cancer patients. Additionally, fresh vegetable juices are an available and economical option that can be used	(Chang et al. 2022)
Gingivitis	Basil		*Ocimum sanctum*	The results of this study showed that the mouthwash prepared from the plant *Ocimum sanctum* had a significant effect on reducing plaque and gingivitis, and this effect was as effective as the use of chlorhexidine in reducing inflammation	(Gupta et al. 2014)
Halitosis	Cranberry Juice		*Vaccinium macrocarpon*	Cranberry ( *Vaccinium macrocarpon* ) has been recommended as a potent natural adjuvant for the prevention of oral diseases, which is due to the anti‐adherence, anti‐cariogenic, and anti‐inflammatory properties of this fruit. Although it has acceptable positive effects in prevention, however, due to the high titratable acidity of cranberry juice (CJ), it may cause gastrointestinal discomfort for consumers and limit the consumption of this drink, which ultimately requires caution in its consumption	(Pellerin et al. 2021a, 2021b)

## 7 Important Points and Recommendations

One of the main concerns of mankind in the fight against infectious diseases is the emergence of bacteria resistant to a wide range of antibiotics. For this reason, the use of an effective and safe alternative method for the prevention and treatment of infectious diseases is considered essential, which can help reduce the consumption of antibiotics and combat bacterial resistance. Since pathological diseases such as periodontal disease, dental plaque, caries, and ultimately halitosis are caused by dysbiosis in the population of resident oral bacteria in the mouth, and the dominance of pathogens over the symbiotic flora leads, as a result, the utilization of probiotics can be a better alternative to antibiotics to reduce gastrointestinal disorders. Identification and selection of probiotic bacteria based on the source of symbiotic microflora in the mouth is probably one of the best strategies for preventing or even treating oral cavity diseases. The various benefits of probiotics, including powerful binding capacity, the formation of protective biolayers, neutralization of acidic pH, antimicrobial activities, modulation of oxidation–reduction potential (ORP), augmentation of the immune system, and reduction of pro‐inflammatory cytokines, can lead to improvement of oral cavity disorders as well as prevention or improvement of halitosis. However, in periodontal diseases, various therapeutic aspects should be considered before using probiotic therapy, including evaluating the length and mode of treatment to prevent a return to a dysbiotic status after treatment; careful screening of cariogenic probiotic strains during periodontal disease treatment; and thorough monitoring and supervision of the probiotic administration in patients with mild to moderate immunosuppression (Karbalaei, Keikha, Kobyliak, et al. 2021).

Although organic and functional foods have potential health benefits, including oral health, it is important to understand the proper use of these products. Excessive consumption of some fruits can cause complex dental consequences, including dental erosion and caries. Dental erosion occurs due to repeated contact of teeth with acidic substances combined with mechanical pressure (Sheibaninia et al. 2014). Despite dental erosion and caries having different histological appearances, the simultaneous occurrence of these two diseases can be harmful to the dental hard tissues. It is necessary for patients to have full knowledge and education about how to consume a variety of fruits and vegetables, herbs, and functional foods. In this regard, Fioravanti et al. (2022) reported that there are correlations between baby nutrition and parents' knowledge of child nutrition with systemic health, oral health, and dental caries. Knowledge and awareness of the parental figure play the most important role for babies to learn to enjoy the flavors of healthy and functional foods. In general, nutrition education during pregnancy and its continuation plays an important role in the path of care as the child grows, and parents' knowledge of child nutrition and their awareness of the interaction between unhealthy sugars in their child's diet and caries formation (Fioravanti et al. 2022). Lifestyle, particularly nutrition is another important and effective factor in oral and dental health and hygiene. Results of an investigation (Mazur et al. 2020) indicated that adult individuals with a plant‐based diet who did not drink beer or wine, spirits, carbonated beverages, and did not consume any highly‐sugared beverages had good overall oral health conditions and exhibited good self‐care and self‐prevention. These features are in agreement with the behavior of these subjects towards an overall healthy lifestyle. Additionally, the results of multiple logistic regression analysis of them showed that fresh fruit consumption at lunch had protective effects on dental caries. Chewing process is critical for normal food intake. Also, the efficiency of chewing a variety of foods needed is decisive in maintaining general health status at all ages (Poli et al. 2021).

Some fruits or plants, such as tangerines ( *Citrus reticulata* ), pineapples ( *Ananas comosus* ), tomatoes, apples, and sour tea ( *Hibiscus sabdariffa* ) are acidic in nature, and most fruit juices, herbal beverages, and vitamin waters contain vitamin C or ascorbic acid, which is acidic. Continuous and improper use can accelerate tooth enamel demineralization. Additionally, excessive consumption of juice from some fruits, including cranberries, can cause digestive upset. As a result, it is recommended that the consumption of fruits and some functional food products (for the prevention or treatment of oral diseases) be in consultation with oral health professionals. The utilization of tooth brushing at least twice a day and avoiding brushing within one hour after consuming acidic foods (Cheng et al. 2009; Lussi et al. 2012; Serre et al. 2016; Stapleton et al. 2012; Surarit et al. 2023).

One of the main concerns of dairy consumers is lactose intolerance, which has created new market opportunities for low‐lactose/lactose‐free dairy foods. Lactose intolerance is caused by the primary malabsorption of lactose. As a result of undigested lactose in the body, lactose is metabolized by the intestinal microflora and is ultimately converted into short‐chain fatty acids like acetate, propionate, butyrate, lactate, and formate, and gases such as hydrogen, methane, and carbon dioxide. Following this, the person may experience symptoms such as bloating, diarrhea, abdominal pain, nausea, and vomiting. One of the treatment options for controlling patients' digestive symptoms is to eliminate milk and dairy products from their diet. This treatment method can lead to serious nutritional deficiencies, such as calcium, phosphorus, and vitamin deficiencies, and then cause damage to the teeth. Current management for people with lactose intolerance involves the use of functional food technologies in the form of replacing regular dairy products with low‐lactose and lactose‐free products, as well as consuming dairy products containing exogenous lactase or probiotics. By increasing consumer awareness of oral health, considering lactose‐intolerant individuals, these patients can be led to choose healthier and safer lactose‐free dairy products. It is also recommended that future research focus on improving the production and development of high‐quality lactose‐free dairy products to provide more options for lactose‐intolerant patients, so that their oral health is not compromised and they can benefit from new products to maintain oral health (Li et al. 2023; Masoumi et al. 2021).

Oral diseases are mainly related to the proliferation of pathogenic bacteria, including *Streptococcus mutans* . Biofilm formation and reactive oxygen species (ROS) production are considered to be the main factors affecting the pathogenicity of bacteria in the oral cavity. These factors create a favorable microenvironment for bacterial growth and provide conditions for the catabolism of substrates and the beginning of teeth's deterioration and oral cancer. Adding antibacterial agents of plant and organic origins to oral care products in the form of functional products can help prevent various diseases. Synthetic antibacterial compounds and their negative effects, as well as the market trend for the use of natural products, have led to the consideration of plant compounds and their components as ingredients in health‐related products. In addition, according to the information analyzed from the results of various studies, it has been found that the essential oils of herbs in dental products, compared to synthetic compounds, have been able to significantly contribute to the sensory acceptance of dental products. Given that the ability to reduce free radicals and inhibit the proliferation of cancer cells are important features of organic and natural compounds, plant‐derived compounds can be considered as an alternative for the development of oral care products that can effectively combat pathogenic bacteria and ROS and are acceptable to consumers. Future research is suggested to evaluate the enrichment of functional products with different plant sources for care and oral hygiene to prove further efficacy (Lugo‐Flores et al. 2021).

## 8 Conclusion and Future Trends

Evidence in the current literature suggests that the consumption of organic foods and foods enriched with phytochemicals from different sources (plant, animal, marine, etc.) provides promising health benefits for different groups of consumers. Organic foods generally contain high and significant levels of various nutrients including minerals, proteins, vitamins, antioxidant compounds, phenols and polyphenols, fatty acids, etc., which, whether consumed raw (fruits and vegetables), extracted or juiced, or isolated and enriched in various food products, have significant positive effects on the prevention and treatment of oral diseases, and the positive effects of these valuable compounds should not be overlooked. Further clinical studies are needed to evaluate the health benefits of foods, beverages, and other manufactured products enriched with organic compounds in the diet of individuals for the control and treatment of oral diseases, as well as their mechanisms of action. Moreover, in the future, more research is needed to achieve more consistent results.

## Author Contributions

**Elahe Aleebrahim‐Dehkordi:** data curation (equal), writing – original draft (equal). **Ahmad Sheibaninia:** writing – original draft (equal), writing – review and editing (equal). **Abdolah Ghasemi Pirbalouti:** conceptualization (lead), investigation (equal), writing – original draft (equal), writing – review and editing (equal).

## Ethics Statement

The authors have nothing to report.

## Consent

The authors have nothing to report.

## Conflicts of Interest

The authors declare no conflicts of interest.

## Data Availability

The data supporting this study's findings are available from the corresponding author upon reasonable request.
